# Carbinolamine Formation and Dehydration in a DNA Repair Enzyme Active Site

**DOI:** 10.1371/journal.pone.0031377

**Published:** 2012-02-22

**Authors:** M. L. Dodson, Ross C. Walker, R. Stephen Lloyd

**Affiliations:** 1 Active Site Dynamics LLC, Houston, Texas, United States of America; 2 San Diego Supercomputer Center and Department of Chemistry and Biochemistry, University of California San Diego, La Jolla, California, United States of America; 3 Center for Research on Occupational and Environmental Toxicology, Oregon Health and Science University, Portland, Oregon, United States of America; Philipps-University Marburg, Germany

## Abstract

In order to suggest detailed mechanistic hypotheses for the formation and dehydration of a key carbinolamine intermediate in the T4 pyrimidine dimer glycosylase (T4PDG) reaction, we have investigated these reactions using steered molecular dynamics with a coupled quantum mechanics–molecular mechanics potential (QM/MM). We carried out simulations of DNA abasic site carbinolamine formation with and without a water molecule restrained to remain within the active site quantum region. We recovered potentials of mean force (PMF) from thirty replicate reaction trajectories using Jarzynski averaging. We demonstrated feasible pathways involving water, as well as those independent of water participation. The water–independent enzyme–catalyzed reaction had a bias–corrected Jarzynski–average barrier height of approximately 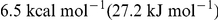 for the carbinolamine formation reaction and 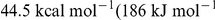) for the reverse reaction at this level of representation. When the proton transfer was facilitated with an intrinsic quantum water, the barrier height was approximately 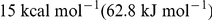 in the forward (formation) reaction and 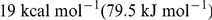 for the reverse. In addition, two modes of unsteered (free dynamics) carbinolamine dehydration were observed: in one, the quantum water participated as an intermediate proton transfer species, and in the other, the active site protonated glutamate hydrogen was directly transferred to the carbinolamine oxygen. Water–independent unforced proton transfer from the protonated active site glutamate carboxyl to the unprotonated N–terminal amine was also observed. In summary, complex proton transfer events, some involving water intermediates, were studied in QM/MM simulations of T4PDG bound to a DNA abasic site. Imine carbinolamine formation was characterized using steered QM/MM molecular dynamics. Dehydration of the carbinolamine intermediate to form the final imine product was observed in free, unsteered, QM/MM dynamics simulations, as was unforced acid-base transfer between the active site carboxylate and the N–terminal amine.

## Introduction

A central tenet of modern biology is the almost completely immutable nature of the genetic information content of DNA. This results from substantial stability over time of both the DNA sequence and structure, despite an enormous variety of physical, chemical, and biological agents capable of compromising its integrity by structurally damaging the DNA. The stability is due, in no small part, to enzymatic DNA repair pathways evolved in response to the selective pressure to maintain this genomic information in an unaltered state, and these repair systems can be categorized by their substrates, structures, and chemical catalytic scenarios. For example, the enzymes of some of these pathways work on the mostly seriously distorting types of DNA damage utilizing enzymes whose structures have been evolutionarily maintained to some degree (e.g., the nucleotide excision repair pathway). Other pathways repair diverse types of base damage with similar catalytic scenarios despite the enzyme proteins themselves being structurally and evolutionarily unrelated. DNA base excision repair (BER) enzymes are of this latter type, and T4 pyrimidine dimer glycosylase is a member of this class [1]. BER enzymes initiate repair by cleaving the nucleotide glycosyl bond (glycosylase step), thereby removing the damaged base (or in some cases inappropriate base, e.g, uracil DNA glycosylase). The damaged DNA substrate specificity for BER enzymes primarily resides in this initial glycosylase step with lesser specificity in subsequent steps. One product of this reaction type is a DNA abasic site that must itself be repaired as it is toxic to the replication and transcriptional activities of the cell [2].

**Figure 1:**
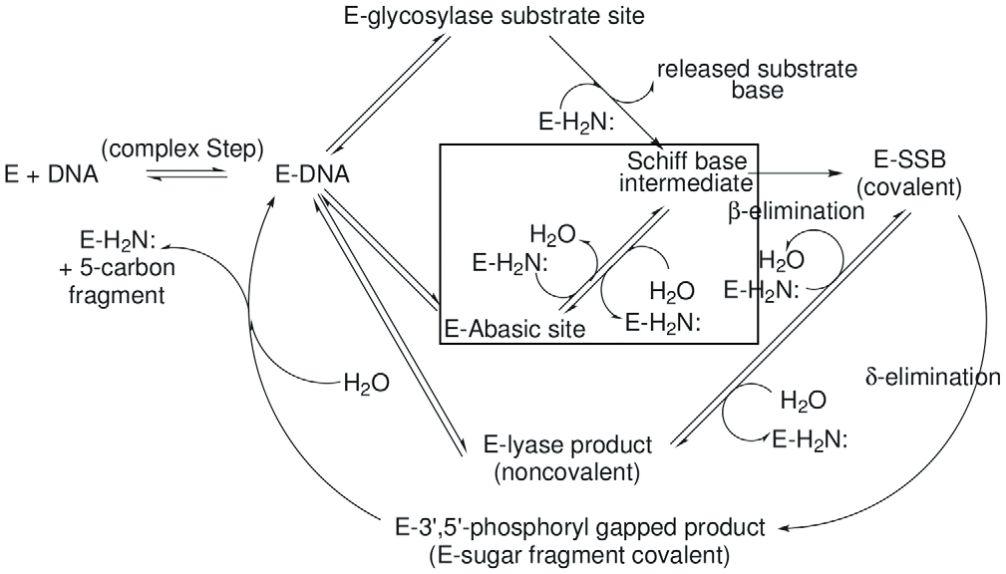
Reactions catalyzed by lyase-capable BER glycosylases. This diagram illustrates the possible reaction pathways catalyzed by these enzymes. The proportions of abasic sites and 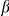
– and 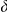
–elimination products in the product spectra depend on the intrinsic chemistry of the imine intermediates and the relative rates of their (1) hydrolytic turnover, e.g., **Schiff base intermediate** 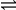 **E–Abasic site** and (2) conversion to “downstream” products, e.g., **Schiff base intermediate** 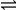 **E–SSB (covalent)**. This report mainly investigates the reactions corresponding to **E–Abasic site intermediate** 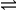 **Schiff base** in the figure: the collapse of the amine nitrogen onto the carbonyl carbon to form the carbinolamine intermediate followed by its dehydration. These reactions are outlined by the rectangular box in the figure. **E–DNA** may represent the enzyme bound at the location of either an abasic site, a pyrimidine photodimer or a lyase product of the 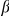
– or 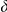
–elimination type.

The various BER enzymes fall into two broad categories based on mechanism: those in which reactions subsequent to glycosyl bond scission may involve lyase–type DNA sugar–phosphate backbone cleavages (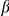
–elimination and, possibly, 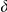
–elimination), and those which leave the DNA backbone intact, e.g., uracil DNA glycosylase. T4PDG initiates repair at pyrimidine ultraviolet photodimers [2], [3] and furnishes an example of the steps, subsequent to glycosyl bond cleavage, characteristic of the first kind of BER enzyme (Fig. 1). The lyase steps, 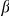
– and 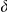
–elimination, proceed via DNA deoxyribose 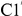
–imine (Schiff base) intermediates in which the amine is the N–terminal threonine 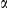
–amine. The protonated imine is thought to act as an electrophilic catalyst [4] for the sequential 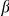
– and 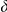
–elimination reactions [2], [3], [5].

In addition to paradigmatic imine intermediates formed at sites of glycosylase reactions, the lyase–capable BER glycosylases can also form identical imines as products of Schiff base formation between the abasic site ring–opened deoxyribose aldehyde and the catalytic active site amine. In Fig. 1, **E-glycosylase substrate site** and **E-abasic site** designate, respectively, Michaelis complexes for the glycosylase and abasic site reactions leading to the key and characteristic **Schiff base intermediate**. This relatively long–lived imine can be reduced by borohydride to form a stable complex suitable for analysis and crystallography, for example see Golan, *et al.* [6] and references therein. In addition to the BER glycosylase/lyase enzymes, reduced imine complexes in other systems featuring covalent enzyme-substrate imine intermediates have been studied by crystallography, e.g., deoxyribose–5–phosphate aldolase [7].

The T4PDG–DNA reduced imine structure 2FCC [6] models some aspects of the product of the glycosylase step and can be compared to 1VAS [8], a structure generally assumed to approximately model the glycosylase Michaelis complex. The protein components of these two structures were shown to be quite similar, as were those parts of the DNA not close to the active site. Differences in the DNA structures in the active sites largely reflected the ring–opened abasic site sugar derivative in 2FCC and the pyrimidine dimer nucleoside with a standard ring–closed deoxyribose moiety in 1VAS. While illustrating some of the structural aspects of enzyme catalysis, analyses of these two structures did not lead to a detailed mechanistic interpretation of the reaction itself, even though they modeled stages before and after the step leading to the key covalent intermediate. Since these enzymes catalyze reactions occurring on time scales of milliseconds to 10s of seconds [9], experimental exploration of mechanisms has been difficult. Most reviews of these enzyme mechanisms invoke speculations extrapolated from studies of model compound reactions in bulk solutions and environments quite different from the active site. Dynamic computational simulations of the reaction based on one or both of the crystal structures may suggest useful mechanistic hypotheses not evident from study of the static structures themselves or easily accessible experimentally. Of course interpreting mechanisms of the pyrimidine dimer glycosylase reaction based on these simulations of its abasic site analog should be confined to homologous reactions in the two systems, e.g., carbinolamine dehydration. Beyond that, these simulations should only be used to suggest hypotheses for the glycosylase reaction.

The initial reaction of an unprotonated amine with an aldehyde forms a Schiff base (imine) carbinolamine. Subsequent dehydration of the carbinolamine yields the final Schiff base product. The **E-Abasic site** 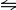 **Schiff base intermediate** step of Fig. 1 is composed of these two sequential reactions. The simulated reactions reported here are enclosed within the rectangular box in Fig. 1. Earlier studies simulating the glycosylase step of T4PDG, based on the 1VAS structure and using a single distance (the glycosylase bond length) as the reaction coordinate, were unsuccessful (unpublished). These simulations showed such chemically unreasonable reactions as sugar C–C bond breakage. Trying to understand these unreasonable results suggested a hypothesis that water might be involved in the glycosylase reaction. Schiff base formation in aqueous solution involves transition states containing water molecules functioning as both nucleophilic and proton transfer reagents [10]. The involvement of water in the deoxyribose–5–phosphate aldolase reaction may be somewhat analogous to the aqueous solution reaction, since a conserved water was observed appropriately positioned to participate in proton transfer steps [7]. However, in the active site of T4PDG, the catalytic Glu22 residue is a possible participant in proton transfer steps [2], [3]. Both the enzyme N–terminal 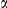
–amine and Glu22 may or may not be protonated, and these two chemical species are the only groups in close proximity to the site of imine formation whose 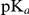 values identify them as likely general acids or bases capable of participating in proton transfer events or in proton exchange with bulk water [6], [11]. Further, Fuxreiter *et al.* [11] have shown that formation of the enzyme–DNA complex shifts the 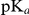 values of active site residues, leading to water penetration of the active site being less favored compared to the unbound enzyme. In turn, this should shift the equilibrium to make the unprotonated amine/protonated carboxylate state of the active site more probable compared to a solvent exposed active site. For purposes of this investigation, the overall charge in the active site was taken to be zero, i.e., the protonation states were either unprotonated amine/protonated carboxylate or protonated amine/unprotonated carboxylate. A natural question then arises from the experimental demonstration [6] of an imine intermediate in the T4PDG reaction: does water participate in the enzyme–catalyzed reactions? A definitive experimental answer cannot be easily obtained, but high quality coupled potential QM/MM simulations of the reaction may lead to insights into this question. At the least such studies might suggest details of similar methods to use when simulating the T4PDG glycosylase reaction. To our knowledge, the involvement of water has not been contemplated in earlier reviews or discussion of experimental or theoretical results for these reactions.

In any QM/MM study, the level of theory for the quantum treatment is of primary importance. DFTB, the Density Functional Theory–based, tight binding Hamiltonian, including the Self Consistent Charge version, SCC–DFTB [12], [13] was used in these investigations [14]–[16]. This quantum treatment, in a QM/MM setting, has been successfully employed to investigate difficult questions of enzyme mechanism [17], [18], and as pointed out by Senn and Thiel [18] “promises, within the validity domain of the parametrization, an accuracy comparable to density functional theory at the cost of semiempirical methods”. It should be mentioned that the system size amenable to *ab initio* molecular dynamics (MD) is very much smaller than that for the QM/MM methods used here. The truncation necessary to reduce the system to a size appropriate for *ab initio* MD would introduce errors and biases requiring extensive preliminary characterizations. These studies would be in computational resource competition with generating increased numbers of replicates for the averaging procedures used in the extraction of potentials of mean force.

Modern molecular dynamics simulation software and the associated force fields and integrators are parameterized such that the effective rate of exploration of phase space is directly comparable to real time. This poses a problem if the time scales of interest are longer than tens of nanoseconds since the simulation computational resource requirements are prohibitive unless the systems are very simple. Several methods have been developed to work around these limitations. Steered MD (SMD) accelerates the time scales of simulations by forcing the trajectory along small subsets of the many degrees of freedom of the complex systems. Since the simulation is forced along these few coordinates and is energetically distorted by this forcing, some means of correcting properties calculated from such trajectories must be used. The Jarzynski relationship [19] allows equilibrium physical properties to be recovered from averages over replicates of nonequilibrium (i.e., directed) analogs of the equilibrium processes [20]–[24].

## Results

Steered MD allows reactions to be simulated that are so improbable on the time scale of molecular dynamics that they would effectively never happen during a simulation. This approach takes advantage of the Jarzynski equality which allows equilibrium properties to be obtained from averages over nonequilibrium processes [25]. Following the approach of Bustamante *et al.* [19], consider a system at temperature 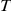 whose equilibrium state is determined by a control parameter 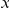. Let the system initially be in state 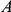 with control parameter 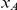. If the system is evolved via a nonequilibrium process by changing 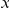 along a given path 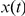 to some final value 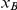, the Jarzynski relationship states that
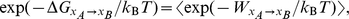
where 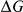 is the free energy difference between equilibrium states 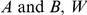 is the work done along the trajectory 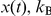 is Boltzmann's constant and 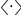 denotes an average over an infinite number of such nonequilibrium experiments repeated under the protocol 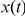. While the derivation of the Jarzynski relationship invokes fairly deep statistical physics, the effect of using Jarzynski exponential averaging for PMF determination along a path is to minimize the impact of higher energy (and, therefore, lower probability) steered trajectories in favor of the lower energy (i.e., more probable) trajectories. The definition of the Jarzynski relationship is as an average over an infinite number of trajectories. Averaging over a finite number of trajectories introduces a truncation bias in the computation. Gore, *et al.* [24] have introduced methods that correct for that bias to a large degree, and have also furnished equations for the mean square errors associated with such a bias–corrected Jarzynski average. These methods have been used in place of a more classical definition of confidence limits. Further details are given in the file S4.

In this study, state 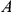 was a snapshot from an equilibrium MD trajectory carried out with a collective progress variable (CV) restrained to its initial value. Multiple such starting structures were obtained by periodically sampling the restrained trajectory. To avoid spurious correlations between SMD replicates started with these snapshots, they were not only obtained from different points in the restrained ensemble trajectory, but also simulated with different values of the pseudo random number generator (PRNG) seed (the system clock in microseconds was used to generate the seed value). The collective variable (a linear combination of interatomic distances) constituted 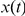, and it was varied from initial to final values under a harmonic restraint. Since in this study covalent bonds were being broken, very stiff restraints were required 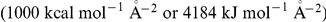. Preliminary studies with restraints in the range of 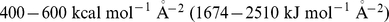 showed that the actual collective variable values achieved sometimes lagged the targeted restrained values. This behavior was followed by discontinuous “jumps” in the CV coordinate as its values “caught up” with the targeted restrained values. These discontinuities complicated the analyses, so 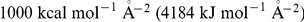 was chosen. This value ensured that the energy of the restraint at all points exceeded the energy of the bonds being broken. Under these conditions, plots of restrained (targeted) CV value versus actual (achieved) CV value were straight lines with slight scatter (governed by the finite width of the underlying harmonic restraint parabola) but no large discontinuities. In all cases, the final value of the CV was attained at the end of the SMD simulations to within an interval consistent with the magnitude of the steering restraint.

**Figure 2:**
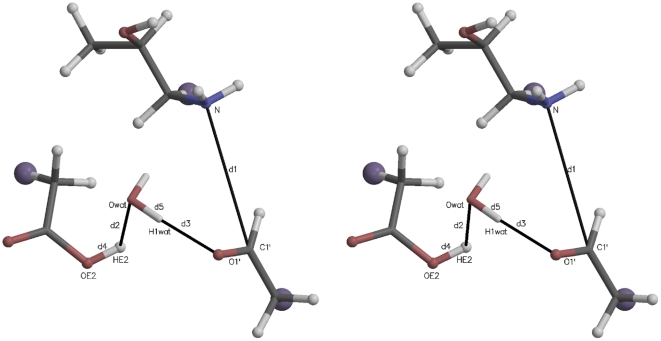
Stereogram of a typical initial quantum region for carbinolamine formation mediated by an active site water. The large purple “atoms” are the link hydrogens, proxies for the classical mechanics sites covalently bonded to the quantum region. The atomic coordinates for this figure were taken directly from one of the Protein Data Bank (PDB) files for the quantum atoms written at the beginning of each QM/MM run. Molecular graphics in this and other similar figures were prepared with MOLSCRIPT [37] and Raster3D [38].

**Figure 3:**
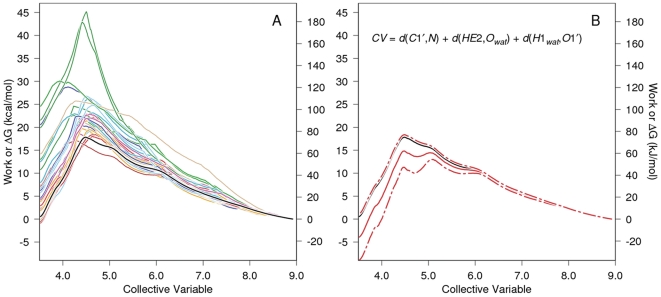
PMF determination for carbinolamine formation with water-mediated proton transfer. The work vs. CV functions for each of 30 SMD replicates are given in color in Panel A as is their Jarzynski average (solid black line). For clarity, only the Jarzynski average, the bias corrected Jarzynski average (solid red line) 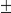 the square root of the mean square error (MSE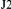 as defined in Gore, *et al.* [24], dashed red lines), and the definition of the CV are shown in Panel B. Note that CV steering is from 8.92 Å to 3.52 Å, i.e., right to left for the formation of the carbinolamine. The large scatter in “barrier height” seen for the individual work functions and the scatter in the work values at the end of the simulation (left axis) are normal and expected for these kinds of methods [39]. Such barrier heights apply only to the pathway followed in that trajectory, and the individual work functions are not good estimates of the pathway 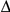
G function for the reaction. The purpose of the exponential averaging in the Jarzynski algorithm is to properly weight the individual work functions so that functions with large peaks contribute only to a degree appropriate to their probability of occurrence. The estimate of 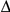
G along the reaction pathway should use the Jarzynski average or some other comparable function such as its bias corrected version (the better choice) [24].

Fig. 2 illustrates the initial active site quantum region structure for the SMD simulation analyzed in Fig. 3. The atom names are those referred to in the collective variable definition in Fig. 3, Panel B. Several distances are also defined: 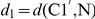, 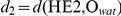, and 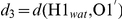, where 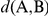 is the distance between atoms 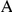 and 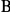. The starting value of the SMD CV was its value at the end of the 1 ns QM/MM relaxation run (8.92 Å). The ending value (3.52 Å) was taken to be the sum of the bond lengths: 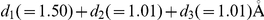. In this and similar simulation setups, the quantum water molecule(s) were restrained to be within a flat well potential spherical volume around 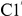 with weak 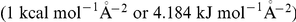 harmonic restraint walls. Further details are given in the file S3.

Fig. 3 shows the PMF determination for carbinolamine formation mediated by an intermediate water acting as a proton transfer reagent. Thirty SMD replicates (colored lines in Panel A) were Jarzynski averaged (solid black line, both panels.) The definition of the elements of Panel B are given in the legend to this figure. The atom names in the CV are identified in Fig. 2.

**Figure 4:**
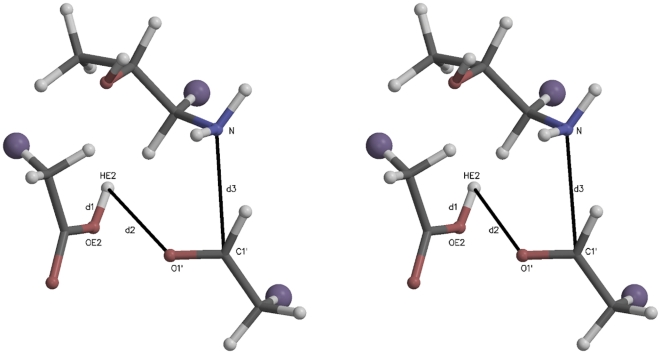
Stereogram of the quantum region for direct proton transfer carbinolamine formation without water involvement. The atom names and distances referenced in Panel B of Fig. 5 are defined. The atoms are colored and the QM/MM region atomic coordinates are as described in Fig. 2.

**Figure 5:**
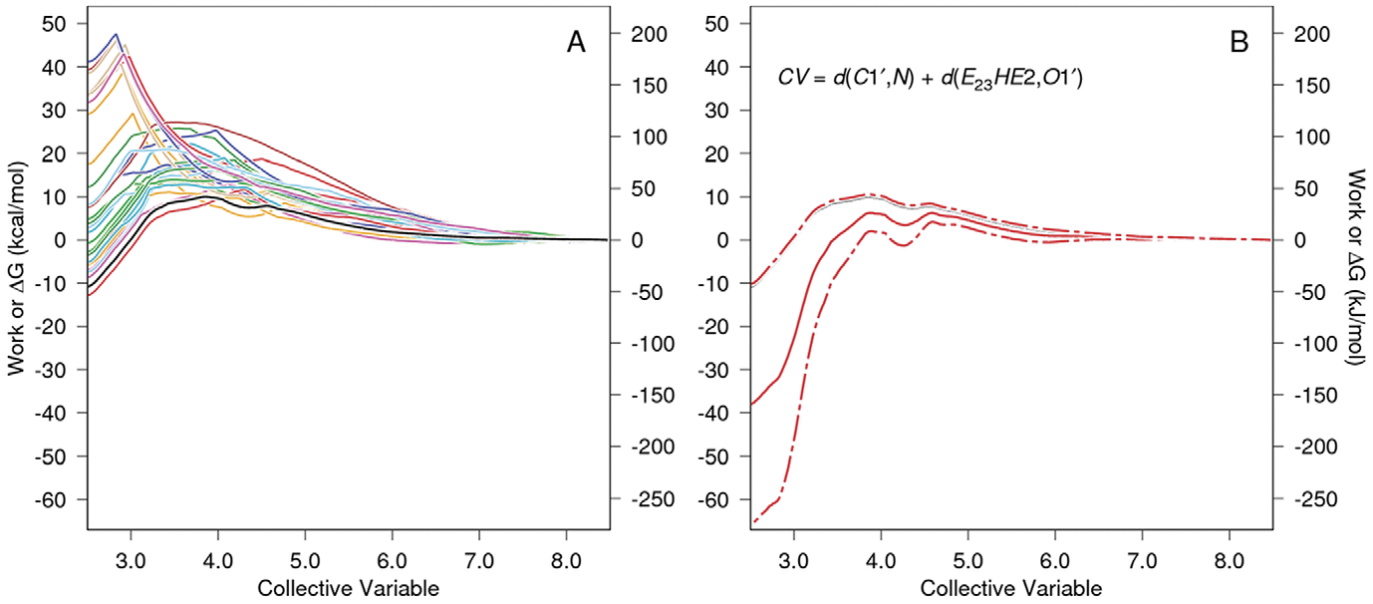
PMF determination for carbinolamine formation by direct proton transfer without involvement of an intermediate water. The work vs. CV functions for each of 30 SMD replicates are given in color in Panel A as is their Jarzynski average (solid black line). For clarity only the Jarzynski average, the bias corrected Jarzynski average 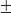 the square root of MSE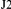, and CV definition are shown in Panel B, and is similar to panel B in Fig. 3. As in that figure, the steered direction for carbinolamine formation is right to left.

Fig. 4 is a stereogram illustrating a typical initial active site quantum region for the SMD simulations further analyzed in Fig. 5 (imine formation without involvement of an intermediate water). The atom names referred to in the CV definition in Fig. 5, Panel B are identified. Interatomic distances are defined similarly to those in Fig. 2.

Fig. 5 shows the PMF determination for direct proton transfer carbinolamine formation without water involvement. Work functions for 30 SMD replicates are shown in Panel A, and atom names referred to in the CV defined in Panel B are identified in Fig. 4. The SMD CV end points were 8.47 Å for the starting value (measured at the end of the 1 ns QM/MM relaxation run) and 2.51 Å at the end: 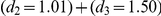 in Fig. 4.

In preparation for SMD studies of imine carbinolamine dehydration, simulations were carried out with a quantum water restrained in the active site by a constant “collective variable”. The starting structure for these simulations was obtained from one of the product structures in the carbinolamine formation study. In these preliminary simulations to generate “snapshots” for SMD, carbinolamine dehydration was sometimes observed without SMD forcing. To follow up on these observations, three simulations were carried out with identical initial structures. In one of the simulations, no dehydration was observed, in a second, dehydration occurred with the participation of the restrained water as an intermediate proton transfer reagent, and in a third, dehydration occurred by direct transfer of the protonated Glu22 hydrogen to the carbinolamine oxygen without participation of the restrained water.

**Figure 6:**
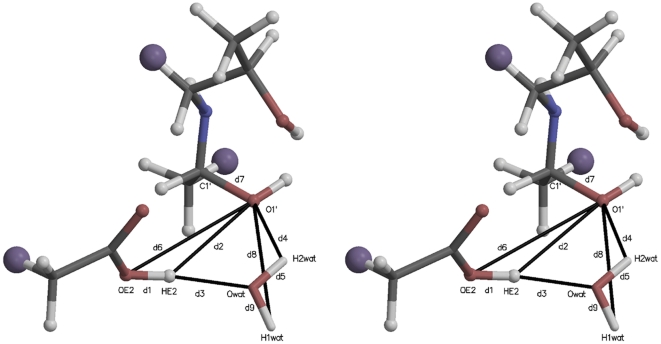
A stereogram for carbinolamine dehydration. As in other such figures, the atom names, link atoms, and key interatomic distances are identified. Note that the structure is that of the imine carbinolamine, not the reactants involved in imine formation.

**Figure 7:**
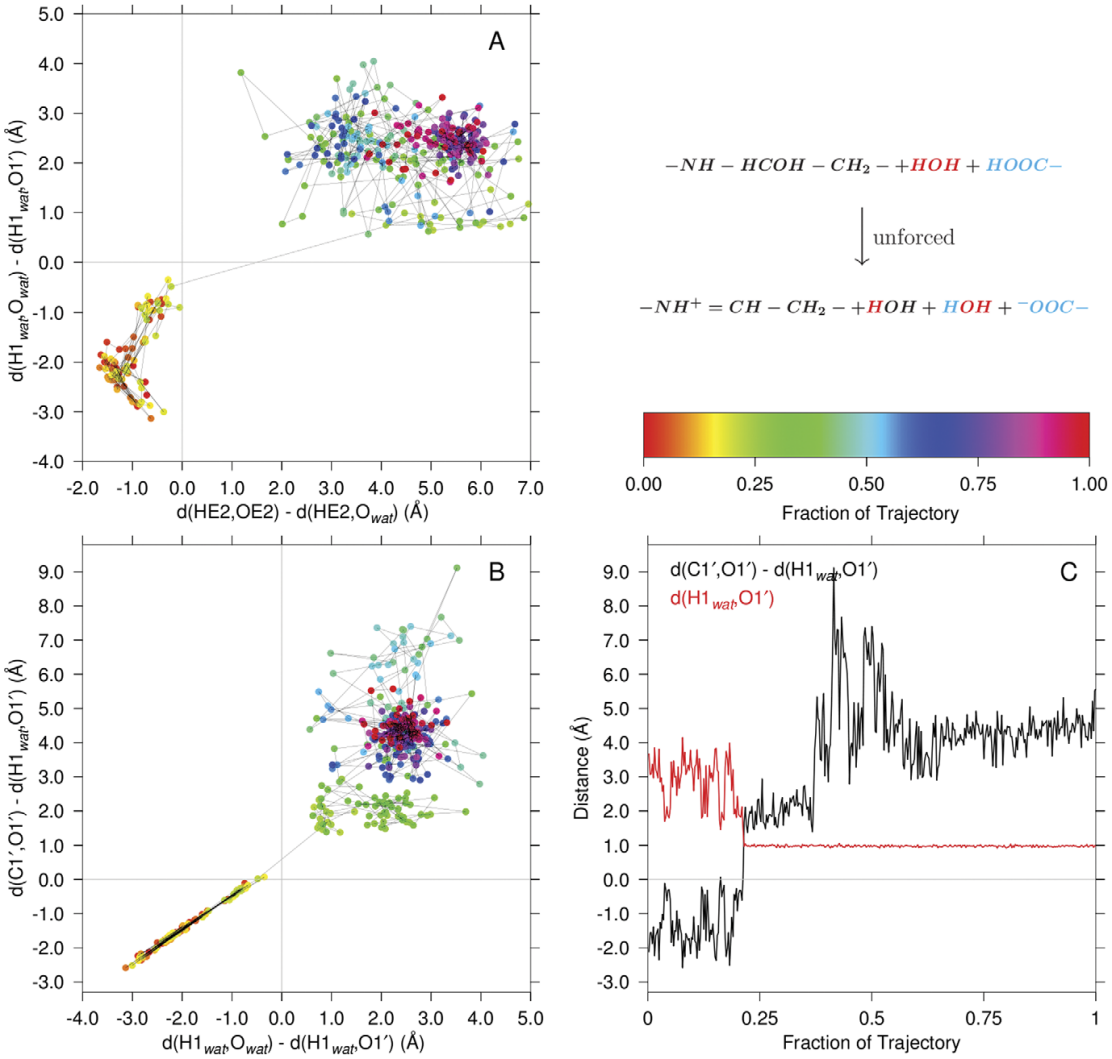
Carbinolamine dehydration with participation of an intermediate water. Panel A shows proton transfers associated with the carbinolamine dehydration in this unforced reaction mediated via a water intermediate. Panel B shows the transfer of the water proton to the carbinolamine oxygen to form the product water. Panel C shows trajectory plots for the oxygen departure (dehydration) and the product 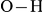 separation (bonding) distance (product water formation). The progress of the simulation is shown color coded as indicated in the palette. The identities of the atoms in quantum region functional groups are shown in color: black, red or blue. The time between samples of the trajectory was 2.5 ps.

**Figure 8:**
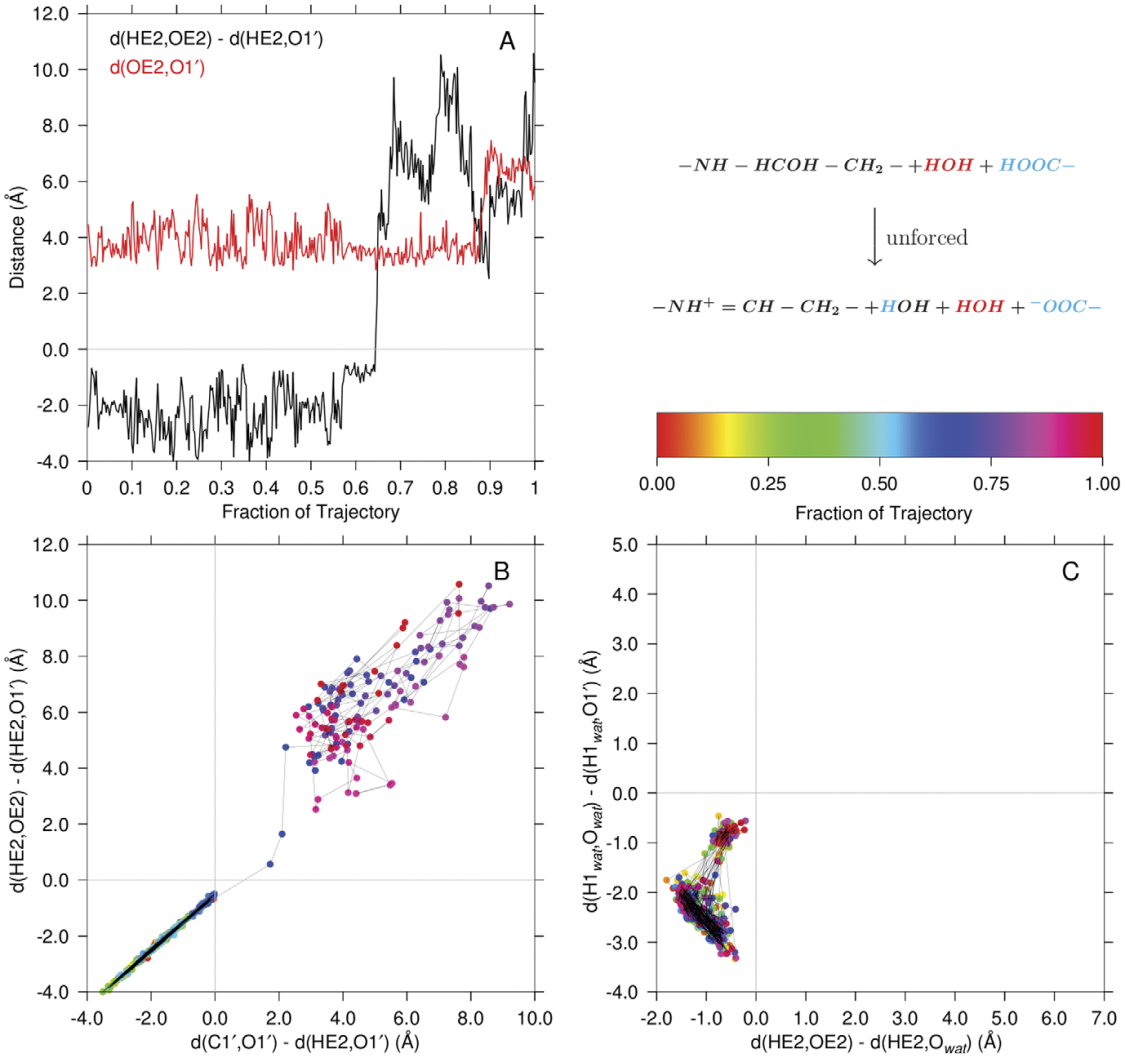
Direct carbinolamine dehydration without involvement of a water acting as a proton transfer reagent. Panel A illustrates the transfer of the protonated glutamate carboxylate hydrogen directly to the carbinolamine hydroxyl oxygen. Shown are the key proton transfer coordinate (black) and the distance between the donor and acceptor oxygens (red) over the trajectory. Panel B compares the 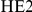 proton transfer (ordinate) to the transfer of 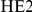 to the 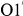 of 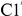 (abscissa) concomitant with 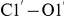 dissociation. The ordinate is the same coordinate shown in black in Panel A: 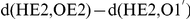. For comparison, Panel C illustrates the trajectory of the third unforced simulation in which carbinolamine dehydration did not occur. The axes in this panel are the same as in Fig. 7, Panel A. The palette shows the progress of the simulation by color coding the data points in panels B and C. The identities of atoms in the quantum region are color coded as in Fig. 7. The time between samples of the trajectory was 2.5 ps.

Fig. 6 is a stereogram of the quantum region for these unforced carbinolamine dehydration simulations, and this diagram corresponds to the active site analyzed in Figs. 7 and 8. As this trajectory was not “steered” between differing endpoints, the CV did not change over its course. The starting (and ending) 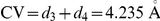. Note that in this kind of restraint, the positions of the individual atoms may change as only the sum of interatomic distances is restrained. The atoms are shaded as in Fig. 2.

In AMBER, residue and atom names do not change over the course of a simulation. At the end of the run analyzed in Fig. 7, the protonated Glu22 carboxylate hydrogen (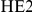) was found associated with the water oxygen (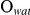) initially present in the active site, as was one of the original water hydrogens (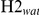), the hydrogen in the restrained CV. In addition, the product water was composed of the other water hydrogen (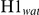), not involved in the restrained CV, the carbinolamine 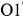 oxygen and 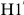 hydrogen. This unambiguously identified the trajectory in Fig. 7 as that of unforced carbinolamine dehydration with the assistance of the initial active site water as an intermediate proton transfer reagent. This trajectory also illustrates that the restraint operates only on the sum of distances: 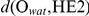 shrank to the equilibrium water 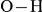 bond length, while 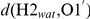 increased to maintain the constant sum.

The axes of Fig. 7, Panel A are proton transfer reaction coordinate variables: 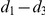 on the abscissa, and 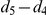 on the ordinate. In these types of proton transfer variables, the value changes from negative to positive as the proton transfers from the donor to the acceptor, with 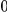 indicating the hydrogen at the midpoint of the donor, acceptor interatomic distance. Progress along the trajectory is coded in color (as defined in the palette). The two proton transfers associated with water-mediated carbinolamine dehydration occur within 2.5 ps of each other, which is the trajectory sampling interval.

The abscissa of Fig. 7, Panel B is the same as the ordinate of Panel A, whereas the ordinate reflects the “transfer of the 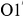 oxygen” (plus its bonded 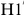 hydrogen) to the unconstrained water hydrogen (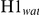), forming the water molecule product of the dehydration reaction. As in Panel A, both reactions occur within the sampling dead time of each other. In this panel, as in Fig. 8, Panel B, a proton transfer coordinate is plotted against the “transfer” of the acceptor oxygen “to a proton” to form water. In the initial part of the trajectory, i.e., in the negative regions of both coordinates, these two movements are seen to be almost completely reciprocal to each other, as reflected in the relatively low scatter in the points.

Panel C shows a trajectory course view of the “transfer of the 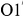 oxygen” (and its bonded 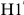 hydrogen) to the water hydrogen to form the water product. The ordinate in this panel is the same as that of Panel B, and measures the oxygen transfer (the dehydration reaction itself). The red curve shows the distance of the water hydrogen to the dehydrating oxygen. Note that the black curve crosses 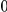 (the oxygen is at the numerical midpoint of the coordinate) at the same time the 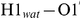 distance becomes equal to and maintains a typical water 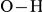 bond length.

In Fig. 8, Panel A, the black line is the distance 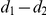 in Fig. 6 and measures proton transfer from the protonated Glu22 carboxylate (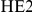) to the carbinolamine oxygen (direct general acid catalyzed carbinolamine dehydration). The red line is 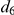, the donor oxygen, acceptor oxygen distance for comparison. The proton transfer, at 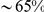 of the trajectory duration, is accompanied by relative stability in the donor-acceptor distance. Eventually, separation of the oxygens due to diffusion diminishes the influence on the proton of the departed carboxylate oxygen at 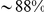 of the trajectory duration. As mentioned above, interrogation of the product structure atom names unambiguously identified the simulated reaction course. Panel B compares the timing of the 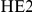 proton transfer from 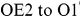 to that of the “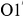 transfer” from 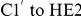. Both reactions occur within one sampling frame.

Fig. 8, Panel C, shows what would be the proton transfer reaction surface as in Fig. 7, Panel A, for a trajectory in which no carbinolamine dehydration was observed. Note the similarity of the dynamics in this trajectory to the initial dynamics in Panel A of Fig. 7 (in the rectangular region bounded by abscissa values of 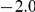 and 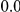, and ordinate values of 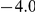 and 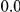).

**Figure 9:**
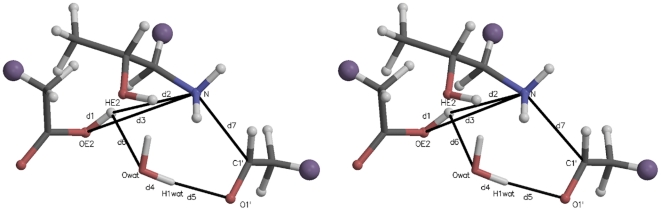
A stereogram for transfer of a proton between a protonated Glu22 carboxylate and an unprotonated N–terminal amine. Atom names are given as in the other similar figures, and the distances involved in the key proton transfer coordinate, 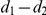, are defined, as is 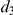, the distance between the donor and acceptor heavy atoms. The atoms are colored as in Fig. 2.

**Figure 10:**
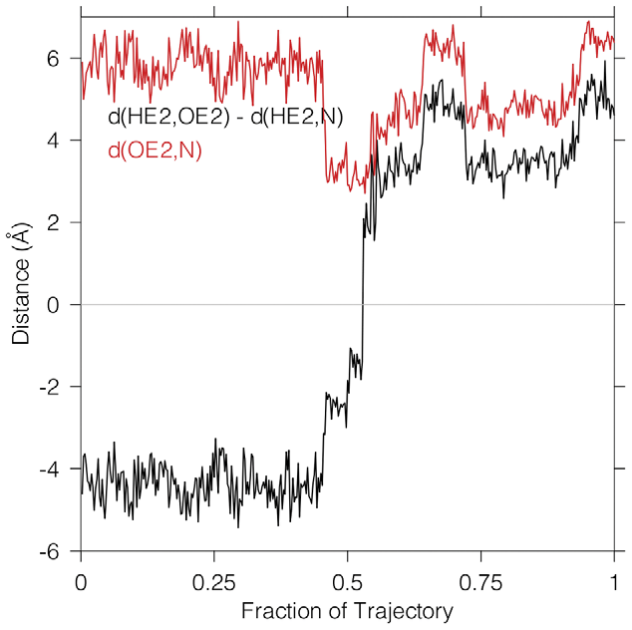
Protonated glutamate carboxyl proton transfer to the N–terminal amine. This graph shows an unforced free dynamics simulation (one of three replicates) in which direct proton transfer between the protonated carboxylate of Glu22 and the unprotonated N–terminal amine nitrogen occurred. The proton transfer coordinate is shown in black, and the donor–acceptor heavy atom distance is shown in red.

In addition to spontaneous dehydration of the carbinolamine, unforced, direct proton transfer of the protonated Glu22 hydrogen to the unprotonated N–terminal amine nitrogen was observed. To investigate this event, Fig. 9 shows the quantum region active site used in three simulations. The structure is that of the carbinolamine formation reactants. All were started from this same structure (which had been observed to undergo this unforced proton transfer event), and with the same restrained quantum water: 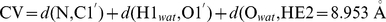 at both the beginning and end of these unforced simulations. Spontaneous proton transfer occurred in one of the three simulations, but without participation of the quantum water. The atom names referred to in the variable definitions in Fig. 10 are given in Fig. 9.

In Fig. 10, the black curve shows the reaction coordinate for the transfer of the HE2 protonated Glu22 hydrogen to the N–terminal amine nitrogen over the course of the trajectory. The proton transfer event occurred slightly after the midpoint of the trajectory duration. The red curve measures the distance between the donor and acceptor heavy atoms, and shows the interatomic distance between them decreasing directly before proton transfer. The potential for spontaneous proton transfer occurred when diffusion of the heavy atoms brought them into close proximity. Subsequent to the proton transfer event, the two curves track each other closely. Two other replicates in this series did not display spontaneous proton transfers of this type, showing only the two proton-heavy atom distances oscillating about their initial values (data not shown).

## Discussion

The question was posed in the introduction whether water might participate in proton transfer reactions in the active site of T4PDG. The PMF determinations in Fig. 3 show a bias–corrected Jarzynski–average barrier height of 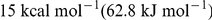 for the forward (carbinolamine formation) and 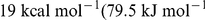) for the reverse reactions when water participates in the reaction as an intermediate proton transfer reagent. Fig. 5 shows a bias–corrected Jarzynski–averaged barrier height of 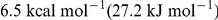 for the forward and 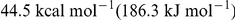 for the reverse reaction when the proton transfer for the forward reaction is directly from the protonated Glu22 general acid to the accepting aldehyde oxygen. The reverse reaction barrier height in Fig. 5 has a large associated uncertainty. This is because of the large work function scatter after (to the left of) the Jarzynski average maximum. A component of the bias correction and MSE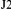 calculation [24] subtracts the Jarzynski average from the the mean of the work functions for all trajectories (this difference is the dissipated work). The large variation in the work functions is manifest in the large square root of the MSE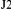 [24] (RMSE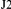.) These two sets of barrier heights are, technically, not directly comparable because of differing definitions of the quantum region in the two types of simulations. Despite this caveat, a speculative interpretation of these differences might imply the greater barrier height of the water-mediated forward reaction involves a significantly smaller probability of correctly aligning the larger number of reactants. The barrier heights for the reverse reactions make them both rather improbable, even taking into consideration the large uncertainty in the reaction without the participation of water. These large barrier heights are consistent with turnover for these reactions occurring on time scales of milliseconds to 10s of seconds [9]. In addition to the scenarios simulated here, other possible general acids acting as proton donors should be considered. Simulations (not shown) of direct proton transfer from the protonated N–terminal amine to the aldehyde oxygen accompanied by carbinolamine formation resulted in very high barriers. However, several other scenarios for this step might be envisioned, e.g., indirect proton transfer from the amine via an intermediate water. This study by no means exhausts these possibilites. Nevertheless, given the approximations in the approach used, and given the observation of unforced direct and water-mediated carbinolamine dehydration reactions (discussed below), carbinolamine formation in the T4PDG active site, both with and without integral proton transfer via an intermediate water, seems feasible. However, the reaction without water would be expected to be more probable. Parenthetically, inclusion of nuclear quantum effects (NQE) would be expected to lower the calculated barrier heights since additional reaction pathways would be made available (e.g., proton tunneling). In general, the exact magnitude of such contributions cannot be predicted *a priori*. Nonetheless, the approach used here should be useful for measuring relative effects, as long as comparisons are only made between similar systems and with the understanding that barrier heights measured without NQE will be overestimated. Of course, if the objectives were calculations leading to comparisons with experimental rates, NQE must be included. However, no such experimental rates were available, and inclusion of NQE was beyond the scope of this project.

The frequent observation of unforced carbinolamine dehydration, with and without water-mediated proton transfer, was unexpected. This implies that the barrier height (at this level of representation) for such reactions would be close to the 300K thermal energy, or there might be no barrier. Since this study did not incorporate NQE [26], no attempt was made to quantitate such small effects. The proton transfer and 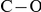 bond cleavage steps in carbinolamine dehydration appear to occur concomitantly within the 2.5 ps trajectory sampling uncertainty. There seems to be no long–lived intermediates in these partial reactions: see Fig. 7 and Fig. 8. Further simulation details are given in the file S3.

The effective 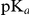 values for the acidic and basic groups within the active site govern the distribution of protonated and unprotonated species. A formal requirement for Schiff base formation is an unprotonated amine, but the possibility of scenarios such as proton transfer from a protonated amine to the aldehyde oxygen via an intermediate water (as mentioned above) makes even this “requirement” more complicated than might otherwise be expected. The possibility of low barrier proton transfer among the acidic and basic groups within the active site has been assumed, and the observation of an unforced version of this proton transfer (at least in the case illustrated by Fig. 10) supports that assumption.

QM/MM simulations of T4PDG-catalyzed reactions at a DNA abasic site revealed possibly significant features of the component carbinolamine formation and dehydration reactions. The PMF for carbinolamine formation was determined by SMD methods with and without participation of water as an intermediate proton transfer reagent. While PMF barrier heights did not completely rule out the feasibility of either scenario, at the level of representation used the barrier height without water involvement was approximately 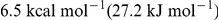 for the forward (formation) reaction and 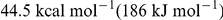 for the reverse reaction. The latter number has a large associated uncertainty. When an active site water served as a proton transfer reagent, the bias–corrected barrier heights were approximately 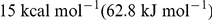 in the forward reaction. The reverse height was 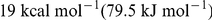. These two cases should not be directly compared in detail since the quantum region definitions differed between them. Imine carbinolamine dehydration was observed to proceed during free QM/MM dynamics via two analogous scenarios: with and without involvement of an intermediate water proton transfer reagent (and without a requirement for SMD forcing in either case). Similar acid–base transfer of the protonated active site Glu22 proton to the N–terminal amine nitrogen was observed in one of three replicate free dynamics simulations. A restrained water was contained in the quantum region during these three simulations, but was not observed participating in covalent bonding changes during the reaction.

In summary, complex proton transfer events, some involving water intermediates, were studied in QM/MM simulations of T4PDG bound to a DNA abasic site. Carbinolamine formation using steering restraints was characterized, and the barrier height for formation was lower in the absence of water. Both the reverse reactions had large barrier heights, and these had large associated uncertainties as measured by the MSE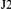 statistic of Gore, *et al.* [24]. In the case of the reaction without an intermediate water proton transfer reagent, the uncertainty was very large. Dehydration of the carbinolamine intermediate to form the final imine product was observed in free, unsteered, QM/MM dynamics simulations, as was unforced acid-base transfer between the active site carboxylate and the N–terminal amine.

Finally, these studies should serve as a reconnaissance for similar investigations of the T4PDG glycosylase reaction, identifying important details necessary for success simulating this more difficult reaction.

## Materials and Methods

The initial structures for these simulations were modeled on the T4PDG, abasic site DNA, reduced imine Protein Data Bank (PDB) entry 2FCC (the A structure was used) [6]. Amino acid residue numbering in this document reflects the processed form of the enzyme protein, i.e., the N–terminal threonine is the second residue in the T4PDG gene sequence, but is referred to here as Thr1 since the various analytical programs in the AMBER suite [27] produce this type of PDB file by default. Details of the computational methods are provided in files S1, S2, and S3 with a synopsis of the most important methods and simulation parameters given here.

### System preparation

New residue descriptors for the unprotonated and *cis* and *trans* protonated forms of the product imine formed between the T4PDG Thr1 amine and the ring–opened abasic site deoxyribose aldehyde, (R)– and (S)–configurations at the 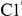 chiral center of the protonated and unprotonated forms of the 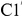 carbinolamines, the ring–opened deoxyribose aldehyde and the unprotonated form of Thr1 were prepared using the AMBER utility program, antechamber [28]. Since in QM/MM simulations these residues would be mostly within the quantum region, the AM1-BCC [29] partial atomic charge calculation scheme was used instead of the more demanding RESP approach [30]. Methods used to prepare the new residue descriptors are available in file S1, and the resulting descriptors and force field additions are available in file S6. Standard AMBER methods, as described in the file S2, were used to equilibrate the density and relax the explicitly solvated DNA-protein complex to the classical MD force field FF99SB [31] and stabilize the temperature at 300K.

### QM/MM simulations

QM/MM simulations were carried out with SHAKE [32] turned off in the quantum region so that proton transfer reactions could be simulated. A 0.5 fs time step was used for the entire calculation to compensate for SHAKE being turned off. The quantum atoms were represented by the DFTB Hamiltonian [12], [15], [16]. The nonbonded cutoff was 9 Å for both regions. The pseudo random number generator seed was derived from the system clock to avoid serial correlations among the trajectories [33]. The electrostatics of the whole system within a periodic boundary representation was handled with a PME treatment of both the classical and quantum regions [13]. The QM/MM relaxation regime always included an additional 1 ns (2 000 000 steps) of dynamics after the system had undergone classical dynamics to allow the system to relax to the coupled potential force field. Temperature and pressure coupling were handled as described in the files S2 and S3, and other simulation details are available in these files.

### Steered molecular dynamics

The AMBER molecular mechanics software suite has had the capability to do SMD using single distance or angle steered coordinates in several recent versions. With AMBER10, a new set of adaptively biased MD (ABMD) and related methods became available. Among other applications, the ABMD subsystem can be used to do SMD. The development of the AMBER version of this methodology has been reported [34], and capabilities to easily specify new ABMD collective variables (CV, also referred to as reaction coordinates) was designed into the software. In collaboration with Dr. Volodymyr Babin (North Carolina State University), the linear combination of distances (LCOD) ABMD variable was implemented to accommodate a requirement for a more flexible distance–based collective variable. LCOD is available as patches to released versions of AMBER9 and AMBER10 and is included in AMBER11 as released.

As an example of an LCOD variable, consider the linear combination of two distances: 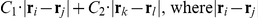 is the distance between atoms 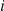 and 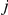. An LCOD variable is specified by a list of atom numbers with an even number of elements and a list of signed real numbers corresponding to the coefficients, 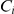, in the linear combination of distances between the atoms taken as pairs. In most cases 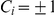. Atom numbers may be repeated as in, for example, 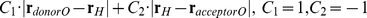, with evolution along the collective variable specified by progress between negative (hydrogen closer to the donor oxygen) and positive (hydrogen closer to acceptor oxygen) limits. This variable would be analogous to, and a generalization of, traditional constrained reaction coordinates for proton transfer between donor and acceptor atoms [35]. The more generalized LCOD variables can also specify more sophisticated reaction progress variables [36] and as used in this communication. Progress along the CV was forced by a harmonic restraint following a defined path. In this study the path was a line segment joining the end points. Since covalent bonds were being formed and broken, large restraint forces  were used.

### Computation of potentials of mean force

Final potentials of mean force (PMF) were calculated by Jarzynski averaging [19]–[24] thirty SMD replicates of 2 000 000 steps (0.5 fs/step, 1 ns) duration. The initial structures for the replicates were prepared by periodically writing “snapshots” during a 1 ns QM/MM simulation carried out as above, except the collective variable was restrained to its initial value with the same harmonic restraint to be used for the SMD runs. These procedures increased the probability that the starting systems for the replicates all sampled the same ensemble. The harmonic restraint energies required to force the steered CV closely along its defined path were Jarzynski averaged, and the results were corrected for truncation bias due to using only a finite number of trajectories in the averages. The MSE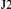 statistic was computed by the methods of Gore, *et al.* [24] as described in more detail in the file S4. The analytical and other software and methods for their use are described in file S5.

## Supporting Information

File S1 Details of new residue descriptors for nonstandard residues in the simulated system. See file S6.
(PDF)

File S2 Details of preliminary molecular dynamics runs to prepare the system for QM/MM simulations.
(PDF)

File S3 Further computational details for the QM/MM simulations.
(PDF)

File S4 A discussion of the Jarzynski average statistical methods of Gore, *et al.* as they apply to the data analysis described here.
(PDF)

File S5 The analytical and other software and their use in this project.
(PDF)

File S6 An archive of the new residue descriptors used in this study and their associated force field modifications.
(BZ2)
