# Supplementary material for: Carbinolamine Formation and Dehydration in a DNA Repair Enzyme Active Site
Source: PLoS One. 2012 Feb 22;7(2):e31377. doi: 10.1371/journal.pone.0031377 (PMC3285167; doi:10.1371/journal.pone.0031377)
Supplement: File S1 — Details of new residue descriptors for nonstandard residues in the simulated system. See file S6. (PDF) [file pone.0031377.s001.pdf]

Supplemental Information for Carbinolamine Formation and Dehydration in a DNA Repair Enzyme Active Site

M. L. Dodson[1,*], Ross C. Walker[2], R. Stephen Lloyd[3]

1 M. L. Dodson, Active Site Dynamics LLC, 611 Chedworth Drive, Houston, TX 77062, USA

2 Ross C. Walker, San Diego Supercomputer Center and Department of Chemistry and Biochemistry, University of California San Diego, La Jolla, CA 92093, USA

3 R. Stephen Lloyd, Center for Research on Occupational and Environmental Toxicology, Oregon Health & Science University, Portland, OR 97239, USA

∗ **E-mail: mldodson@comcast.net**

New residue descriptors

Before MD simulations of molecules containing nonstandard residues for the chosen force field could be carried out, descriptors for the new residue types were prepared using the Assisted Model Building with Energy Refinement, version 10 [1] (AMBER10) utility program antechamber to assign partial atomic charges and atom types consistent with the AMBER FF99SB force field definition [1]. The simulations carried out in this study required several of these new residues to be constructed (although ultimately not all were found to be required): (1) Unprotonated and *cis* and *trans* protonated forms of the product imine formed between the T4PDG N–terminal threonine amine and the ring–opened abasic site deoxyribose aldehyde. Although both *cis* and *trans* forms of the protonated imine were prepared, it was found that only the *trans* version fit within the enzyme active site. (2) (R)– and (S)–configurations at the C1′ chiral center of the protonated and unprotonated forms of the C1′ carbinolamines corresponding to the imines were prepared. Preliminary simulations of carbinolamine formation identified the (R)–carbinolamine as the preferred product. Finally, the following reactant residue descriptors were prepared as described above: (3) the ring–opened deoxyribose aldehyde and (4) the unprotonated form of the N–terminal threonine. The AMBER force field atom types were assigned by analogy to the FF99SB force field, and the partial atomic charges were calculated within antechamber using the AM1-BCC method [2]. Since the QM/MM simulations encompassed the nonstandard residue atoms within the quantum region, the descriptor charges and valence parameters were only used during the preliminary classical MD relaxation

steps prior to the QM/MM reaction simulations. Therefore, the more computationally complex RESP [3] charge fitting procedure was not used. The new residue descriptors have been uploaded as supplemental data within the compressed tape archive file: S6.tar.bz2. This compressed archive can be unpacked with the tar utility on recent Mac or Unix operating systems. For older operating system versions, it may be necessary to use the bunzip2 utility before expanding the archive with the tar utility. The appropriate utility programs should be available or easily obtainable at any facility with a recent AMBER installation. Windows users can unpack the archive with the free 7-Zip utility.

References

1. Case D, Darden T, TE Cheatham I, Simmerling C, Wang J, et al. (2008) AMBER 10. The University of California, San Francisco.

2. Jakalian A, Bush BL, Jack DB, Bayly CI (2000) Fast, efficient generation of high–quality atomic charges. AM1–BCC model: I. Method. J Comput Chem 21: 132–146.

3. Cornell WD, Cieplak P, Bayly CI, Kollmann PA (1993) Application of RESP charges to calculate conformational energies, hydrogen bond energies, and free energies of solvation. J Amer Chem Soc 115: 9620–9631.
