# Supplementary material for: Carbinolamine Formation and Dehydration in a DNA Repair Enzyme Active Site
Source: PLoS One. 2012 Feb 22;7(2):e31377. doi: 10.1371/journal.pone.0031377 (PMC3285167; doi:10.1371/journal.pone.0031377)
Supplement: File S2 — Details of preliminary molecular dynamics runs to prepare the system for QM/MM simulations. (PDF) [file pone.0031377.s002.pdf]

Supplemental Information for Carbinolamine Formation and Dehydration in a DNA Repair Enzyme Active Site

M. L. Dodson[1,*], Ross C. Walker[2], R. Stephen Lloyd[3]

1 M. L. Dodson, Active Site Dynamics LLC, 611 Chedworth Drive, Houston, TX 77062, USA

2 Ross C. Walker, San Diego Supercomputer Center and Department of Chemistry and Biochemistry, University of California San Diego, La Jolla, CA 92093, USA

3 R. Stephen Lloyd, Center for Research on Occupational and Environmental Toxicology, Oregon Health & Science University, Portland, OR 97239, USA

∗ **E-mail: mldodson@comcast.net**

System preparation

The parameter–topology (prmtop) and coordinate (coord) simulation input files were created with AMBER10 utility programs tleap or xleap using FF99SB [1]. The 2FCC Protein Data Bank (PDB) file [2], structure "A", was taken as the initial model. The initial protonation state of the active site glutamate, Glu22, was assigned during the prmtop/coord file preparation and was varied according to the simulation to be carried out. The simulation system consisted of the solute (protein and DNA) and a truncated octahedral explicit solvent periodic boundary system consisting of approximately 10 000 TIP3P [3] water molecules (the exact number varying with the system being simulated) and sodium ions to achieve electrical neutrality.

The system was prepared for simulation with a series of minimizations and MD simulations to repair possible structural imperfections that might have been introduced by taking the 2FCC reduced imine PDB entry as the basis of the simulation structures and to equilibrate the system temperature and density: An initial 1000 step minimization was carried out using a 1 kcal $\text{mol}^{-1}\text{Å}^{-2}$ (4.184 kJ $\text{mol}^{-1}\text{Å}^{-2}$) harmonic solute restraint to relax the waters. This was followed by a 12 500 step (2 fs/step, 25 ps) constant volume simulation, ramping the temperature (using the Berendsen weak–coupling algorithm [4]) from 100K to 300K in 500 steps (1 ps), followed by 12 000 steps (24 ps) at 300K. The solute was subject to a 1 kcal $\text{mol}^{-1}\text{Å}^{-2}$ (4.184 kJ $\text{mol}^{-1}\text{Å}^{-2}$) harmonic restraint during this run. This was followed by 12 500 steps (25 ps) of (solute restrained) constant pressure periodic boundary conditions (PBC)

particle mesh Ewald (PME) MD [5] to equilibrate the density. Next, six 1000 step minimization cycles with the solute restraint relaxed from 25 to 0 kcal $mol^{-1}Å^{-2}$ (104.6 kJ $mol^{-1}Å^{-2}$ to 0 kJ $mol^{-1}Å^{-2}$) in 5 kcal $mol^{-1}Å^{-2}$ (20.9 kJ $mol^{-1}Å^{-2}$) steps were carried out. After minimization, the system was warmed to 300K with Langevin dynamics using a gamma_ln (collision frequency) parameter of 1 ps^{-1} for 100 000 steps of 2 fs/step dynamics (200 ps). Finally, the system underwent 500 000 steps (2 fs/step, 1 ns) of constant volume (NVE) dynamics. The temperature was stable at this stage. All the preliminary molecular dynamics relaxation steps used only the classical FF99SB force field, and the lengths of bonds involving hydrogen were constrained with the SHAKE algorithm [6]. The nonbonded cutoff was set at 9 Å. QM/MM simulations were carried out using the full interaction calculations for all atoms, including hydrogens, so that proton transfer reactions could be simulated. A 0.5 fs time step was used for the entire calculation.

References

1. Case D, Darden T, TE Cheatham I, Simmerling C, Wang J, et al. (2008) AMBER 10. The University of California, San Francisco.

2. Golan G, Zharkov DO, Grollman AP, Dodson ML, McCullough AK, et al. (2006) Structure of T4 pyrimidine dimer glycosylase in a reduced imine covalent complex with abasic site–containing DNA. J Mol Biol 362: 241–258.

3. Jorgensen W, Chandrasekhar J, Madura J, Impey R, Klein M (1983) Comparison of simple potential functions for simulating liquid water. J Chem Phys 79: 926–935.

4. Berendsen HJC, Postma JPM, van Gunsteren WF, DiNola A, Haak JR (1984) Molecular dynamics with coupling to an external bath. J Chem Phys 81: 3684–3690.

5. Darden T, York D, Pedersen L (1993) Particle mesh Ewald: An N·log(N) method for Ewald sums in large systems. J Chem Phys 98: 10089–10092.

6. Ryckaert JP, Ciccotti G, Berendsen HJC (1977) Numerical integration of the Cartesian equations of motion of a system with constraints: Molecular dynamics of n–alkanes. J Comput Phys 23: 327–341.
