# Supplementary material for: Carbinolamine Formation and Dehydration in a DNA Repair Enzyme Active Site
Source: PLoS One. 2012 Feb 22;7(2):e31377. doi: 10.1371/journal.pone.0031377 (PMC3285167; doi:10.1371/journal.pone.0031377)
Supplement: File S3 — Further computational details for the QM/MM simulations. (PDF) [file pone.0031377.s003.pdf]

Supplemental Information for Carbinolamine Formation and Dehydration in a DNA Repair Enzyme Active Site

M. L. Dodson[1,*], Ross C. Walker[2], R. Stephen Lloyd[3]

1 M. L. Dodson, Active Site Dynamics LLC, 611 Chedworth Drive, Houston, TX 77062, USA

2 Ross C. Walker, San Diego Supercomputer Center and Department of Chemistry and Biochemistry, University of California San Diego, La Jolla, CA 92093, USA

3 R. Stephen Lloyd, Center for Research on Occupational and Environmental Toxicology, Oregon Health & Science University, Portland, OR 97239, USA

∗ **E-mail: mldodson@comcast.net**

QM/MM simulations

The QM/MM simulations were carried out using the Purdue University Steele cluster resource on the TeraGrid and were supported by the NSF. Typical run times were 96–97 hr for a 2 000 000 step (0.5 fs/step, 1 ns) simulation.

General features of the QM/MM dynamics simulations included the following: (1) the temperature was maintained at 300K with Langevin dynamics, gamma_ln = 2 ps^{-1}; (2) the complete bond interactions, including hydrogens, were calculated in the force evaluation; (3) the nonbonded cutoffs in the QM and classical regions were set to 9 Å; (4) the pseudo random number generator was reseeded with a new integer at every restart to avoid serial correlations among the trajectories [1]; (5) the QM method used was DFTB; and (6) the electrostatics of the whole system were handled with a full PME treatment [2]. Before production simulations, 2 000 000 steps of 0.5 fs/step QM/MM dynamics (1 ns) were carried out to relax the quantum and surrounding classical regions to the coupled potential force field. Restart files for steered molecular dyanamics (SMD) were prepared by selecting "snapshots" from a subsequent 2 000 000 step (0.5 fs/step, 1 ns) simulation. This simulation was carried out with the collective variable (CV) restrained to its initial value with the same restraint used for the SMD replicates. This initial CV value was taken to be the appropriate sum of distances observed in the last frame of the QM/MM relaxation run. The final CV value was taken to be the sum of the appropriate equilibrium bond lengths. These procedures were used to increase the probability that the initial systems for the SMD replicates sampled

the same ensemble.

To keep water molecules within the active site, water oxygens were restrained with a flat well, one-sided harmonic potential centered at C'_1. The water oxygen restraint was 1 kcal $mol^{-1}Å^{-2}$ (4.184 kJ $mol^{-1}Å^{-2}$). This restraint results in waters remaining within a spherical cavity appoximately encompassing the quantum region. The flat well nature of the restraint implies water molecules within the restraint distance (within the sphere) were not influenced by the restraining force. Since inclusion of waters not involved in the collective variable (CV) complicated the definition of the quantum region, later simulations (those reported here) only restrained waters named in the CV. The flat well potential restraint was maintained for these waters, but naming them in the CV was probably a sufficient restraint since the CV kept the waters well within the flat well portion of the restraint.

References

1. Cerutti DS, Duke R, Freddolino PL, Fan H, Lybrand TP (2008) A vulnerability in popular molecular dynamics packages concerning Langevin and Andersen dynamics. J Chem Theory Comput 4: 1669–1680.

2. Walker RC, Crowley MF, Case DA (2008) The implementation of a fast and accurate QM/MM potential method in Amber. J Comput Chem 29: 1019–1031.
