# Supplementary material for: Carbinolamine Formation and Dehydration in a DNA Repair Enzyme Active Site
Source: PLoS One. 2012 Feb 22;7(2):e31377. doi: 10.1371/journal.pone.0031377 (PMC3285167; doi:10.1371/journal.pone.0031377)
Supplement: File S4 — A discussion of the Jarzynski average statistical methods of Gore, et al. as they apply to the data analysis described here. (PDF) [file pone.0031377.s004.pdf]

Supplemental Information for Carbinolamine Formation and Dehydration in a DNA Repair Enzyme Active Site

M. L. Dodson[1,*], Ross C. Walker[2], R. Stephen Lloyd[3]

1 M. L. Dodson, Active Site Dynamics LLC, 611 Chedworth Drive, Houston, TX 77062, USA

2 Ross C. Walker, San Diego Supercomputer Center and Department of Chemistry and Biochemistry, University of California San Diego, La Jolla, CA 92093, USA

3 R. Stephen Lloyd, Center for Research on Occupational and Environmental Toxicology, Oregon Health & Science University, Portland, OR 97239, USA

∗ **E-mail: mldodson@comcast.net**

Calculation of the Gore *et al.* statistics [1, 2]

Following the approach of Bustamante *et al.* [1,2], consider a system at temperature T whose equilibrium state is determined by a control parameter x. Let the system initially be in state A with control parameter x_A. If the system is evolved via a nonequilibrium process by changing x along a given path $x(t)$ to some final value x_B, the Jarzynski relationship states that

$$\exp(-\Delta G_{x_A \to x_B}/k_\mathrm{B}T) = \langle \exp(-W_{x_A \to x_B}/k_\mathrm{B}T) \rangle,$$

where ΔG is the free energy difference between equilibrium states A and B, W is the work done along the trajectory $x(t)$, k_B is Boltzmann's constant and $\langle \cdot \rangle$ denotes an average over an infinite number of such nonequilibrium experiments repeated under the protocol $x(t)$. Alternatively, the Jarzynski average is an estimate for the path $x(t)$ traversed at infinitely slow velocity, i.e., by a quasi-static transformation [3, p. 339]. To calculate a full estimate of the potential of mean force over a reaction, a Jarzynski average must be calculated at each trajectory sampling time x_j. It should be emphasized that the Jarzynski relationship is defined by an average over an infinite number of trajectories. Averaging over only a finite number of trajectories introduces a truncation bias. Gore, *et al.* have reported methods to correct for this bias. The calculation of the finite Jarzynski average for N replicates and one trajectory sample j is

given by:

$$\Delta \hat{G}_J(N) = -\beta^{-1}\log\left[\frac{1}{N}\sum_{i=1}^{N} e^{-\beta W_i}\right],$$

where

$$\beta = \frac{1}{k_{\mathrm{B}}T}.$$

The mean work is calculated as:

$$\Delta \hat{G}_{MW}(N) = \langle W\rangle_N = \frac{1}{N}\sum_{i=1}^{N} W_i.$$

Dropping the N for clarity, the dissipative work is given by:

$$\bar{W}_{dis} = \langle W\rangle - \Delta \hat{G}_J.$$

The bias and mean square error (MSE) require a function, $\alpha(\bar{W}_{dis})$, which depends on a parameter C (Gore, *et al.* equation 9):

$$\alpha = \frac{\log[2\beta C \bar{W}_{dis}]}{\log[C(e^{2\beta \bar{W}_{dis}} - 1)]}.$$

A sensitivity analysis demonstrated that the α function showed only minor differences for $C = 30$, 40 or 50, whereas $C = 15$ deviated to a larger degree from the others. $C = 40$ seems a good compromise.

A first order correction to the dissipative work is given by (sentence before Gore, *et al.* equation 19):

$$\hat{\bar{W}}_{dis2} = \langle W\rangle - \Delta \hat{G}_J + \frac{\hat{\bar{W}}_{dis}}{N^{\alpha(\hat{\bar{W}}_{dis})}},$$

and the final free energy difference estimator is given by Gore, *et al.* equation 19. This is the bias–corrected Jarzynski average:

$$\Delta \hat{G}_{J2} = \Delta \hat{G}_J - \hat{B}_{J2},$$

where

$$\hat{B}_{J2} = \frac{\hat{\bar{W}}_{dis2}}{N^{\alpha(\hat{\bar{W}}_{dis2})}}.$$

The MSE estimator is given by Gore, *et al.* equation 17,

$$\mathrm{MSE}_J = \frac{2\bar{W}_{dis}}{\beta N^{\alpha(\bar{W}_{dis})}} + \frac{(\bar{W}_{dis})^2}{(N^{\alpha(\bar{W}_{dis})})^2},$$

and for the $J2$ estimator (used here):

$$\mathrm{MSE}_{J2} = \frac{2\bar{W}_{dis2}}{\beta N^{\alpha(\bar{W}_{dis2})}} + \frac{(\bar{W}_{dis2})^2}{(N^{\alpha(\bar{W}_{dis2})})^2}.$$

The RMSE_{J2} is given by $\mathrm{sqrt}(\mathrm{MSE}_{J2})$.

References

1. Gore J, Ritort F, Bustamante C (2003) Bias and error in estimates of equilibrium free–energy differences from nonequilibrium measurements. Proc Nat Acad Sci USA 100: 12564–12569.

2. Bustamante C, Liphardt J, Ritort F (2005) The nonequilibrium thermodynamics of small systems. Physics Today 58, Iss. 7: 43–48.

3. Xiong H, Crespo A, Marti M, Estrin D, Roitberg A (2006) Free energy calculations with non–equilibrium methods: Applications of the Jarzynski relationship. Theoretical Chemistry Accounts: Theory, Computation, and Modeling (Theoretica Chimica Acta) 116: 338–346.
