# Supplementary material for: Carbinolamine Formation and Dehydration in a DNA Repair Enzyme Active Site
Source: PLoS One. 2012 Feb 22;7(2):e31377. doi: 10.1371/journal.pone.0031377 (PMC3285167; doi:10.1371/journal.pone.0031377)
Supplement: File S5 — The analytical and other software and their use in this project. (PDF) [file pone.0031377.s005.pdf]

Supplemental Information for Carbinolamine Formation and Dehydration in a DNA Repair Enzyme Active Site

M. L. Dodson[1,*], Ross C. Walker[2], R. Stephen Lloyd[3]

1 M. L. Dodson, Active Site Dynamics LLC, 611 Chedworth Drive, Houston, TX 77062, USA

2 Ross C. Walker, San Diego Supercomputer Center and Department of Chemistry and Biochemistry, University of California San Diego, La Jolla, CA 92093, USA

3 R. Stephen Lloyd, Center for Research on Occupational and Environmental Toxicology, Oregon Health & Science University, Portland, OR 97239, USA

∗ **E-mail: mldodson@comcast.net**

Analytical and other software

Most analyses described here were carried out with Perl scripts developed in-house. The statistics described by Gore, *et al.* [1] were calculated as described in file S4.pdf. As a final step, many of the Perl scripts wrote programs in the Gri graphics language (Gri language for scientific illustration; Dan Kelley, Department of Oceanography, Dalhousie University, Halifax, NS B3H 4J1). The Gri programs generated the graphs in the body of the text. These Perl scripts will be made available upon emailed request, but with no further support. Molecular graphics were prepared with MOLSCRIPT [2] and Raster3D [3] directly from PDB files of the quantum region written by AMBER. The orientation matrices for the desired view of the quantum region were generated with VMD [4].

References

1. Gore J, Ritort F, Bustamante C (2003) Bias and error in estimates of equilibrium free–energy differences from nonequilibrium measurements. Proc Nat Acad Sci USA 100: 12564–12569.

2. Kraulis PJ (1991) MOLSCRIPT: A program to produce both detailed and schematic plots of protein structures. Journal of Applied Crystallography 24: 946–950.

3. Merritt EA, Bacon DJ (1997) Raster3D: Photorealistic molecular graphics. Methods in Enzymology 277: 505–524.

4. Humphrey W, Dalke A, Schulten K (1996) VMD–Visual molecular dynamics. J Molec Graphics 14: 33–38.
